# Wang-Bi Capsule Alleviates the Joint Inflammation and Bone Destruction in Mice with Collagen-Induced Arthritis

**DOI:** 10.1155/2020/1015083

**Published:** 2020-03-19

**Authors:** Hua Cui, Haiyang Shu, Dancai Fan, Xinyu Wang, Ning Zhao, Cheng Lu, Aiping Lu, Xiaojuan He

**Affiliations:** ^1^Institute of Basic Research in Clinical Medicine, China Academy of Chinese Medical Sciences, Beijing 100700, China; ^2^School of Life Science and Engineering, Southwest Jiaotong University, Chengdu 611756, China; ^3^The Second Clinical College of Guangzhou University of Chinese Medicine, Guangzhou 510006, China; ^4^Law Sau Fai Institute for Advancing Translational Medicine in Bone & Joint Diseases, School of Chinese Medicine, Hong Kong Baptist University, Kowloon Tong, Hong Kong

## Abstract

Wang-Bi Capsule (WB), a traditional Chinese medicine- (TCM-) based herbal formula, is currently used in clinic for the treatment of rheumatoid arthritis (RA) with positive clinical effects. However, its pharmacological mechanism of action in RA is still obscure. Therefore, this study established a collagen-induced arthritis (CIA) mice model to examine the efficacy of WB by using arthritis score, histological analysis, and micro-CT examination. Proinflammatory cytokines expression, osteoclast number, OPG/RANKL system, and NF-*κ*B activation were then detected to further investigate the mechanism of WB in RA treatment. The results indicated that WB could alleviate the erythema and swelling of paws in CIA mice. It also inhibited the infiltration of inflammatory cells and bone destruction and increased bone density in joints of CIA mice. Mechanistic studies showed that WB treatment decreased the production of IL-1*β*, IL-6, and TNF-*α* in serum and joints of CIA mice. Moreover, it reduced the osteoclast number, increased OPG level, decreased RANKL level, and inhibited the activation of NF-*κ*B in joints of CIA mice. In conclusion, this study demonstrated that WB could effectively alleviate disease progression of CIA mice by decreasing the IL-1*β*, IL-6, and TNF-*α* levels, modulating the OPG/RANKL system, and inhibiting the activation of NF-*κ*B.

## 1. Introduction

Rheumatoid arthritis (RA) is a complex, chronic inflammatory disease with approximately 0.24% global prevalence [1]. The pathophysiology of RA is characterized by a variety of immune cells with aberrant inflammatory cytokines such as TNF-*α*, IL-1, IL-6, and IL-17, infiltrating in the synovium of multiple joints, and those inflammatory mediators lead to long-term inflammation and the formation of pannus, which ultimately result in irreversible joint and cartilage destruction and sever disability [2, 3]. Currently, the purpose of RA treatment is mainly to reduce the inflammatory response, inhibit the development of lesions and bone damage, and protect the function of joints. Up to now, it is still difficult to find an ideal drug to completely cure this disease. Current anti-inflammatory drugs, no matter nonsteroidal anti-inflammatory drugs (NSAIDs) or steroid or biological response modifiers, could not meet the need of all RA patients.

Traditional Chinese medicine (TCM) has attracted more and more attention due to its advantages of safety and less adverse reactions. Wang-Bi Capsule (WB), a TCM-based herbal formula, has been used to treat RA in China for several years and shown positive clinical effects. It is composed of seventeen herbal medicines: *Rehmannia glutinosa* Libosch., *Rehmannia glutinosa (Gaetn.)* Libosch. ex Fisch. et Mey., *Dipsacus asper* Wall. ex Henry, *Aconitum carmichaeli* Debx., *Angelica pubescens* Maxim. f. biserrata Shan et Yuan, *Drynaria fortunei* (Kunze) J. Sm., *Cinnamomum cassia* Presl, *Epimedium brevicornu* Maxim., *Saposhnikovia divaricata* (Trucz.) Schischk., *Clematis chinensis* Osbeck, *Gleditsia sinensis* Lam., *Capra hircus* Linnaeus, *Paeonia lactiflora* Pall., *Ciborium barometz* (L.) J. Sm., *Anemarrhena asphodeloides* Bunge, *Lycopodium japonicum* Thunb., *Carthamus tinctorius* L. Recently, the pharmacological effects of some components from WB in RA have been found. Zhang and Dai reported that total paeoniflorin, the major active component of *Paeonia lactiflora* Pallas, could inhibit the proliferation of lymphocytes and fibroblast-like synovium cells, and the production of matrix metalloproteinases [4]. Chi et al. indicated that icariin, isolated from the *Epimedium* family, decreased Th17 cells and the production of IL-17 through inhibiting STAT3 activation in collagen-induced arthritis (CIA) mice [5]. Kong et al. showed that *Saposhnikovia divaricata* chromone extract (SCE) reduced the protein level of NF-*κ*B and inhibited p-ERK, p-JNK, and p-p38 expression in human fibroblast-like synoviocytes and CIA rats [6]. However, the pharmacological mechanism of action of WB in RA is still obscure. In this study, we want to determine the effect of WB in a CIA mouse model and understand its mechanism of action.

## 2. Materials and Methods

### 2.1. Animals

Male DBA/1J mice (6–8 weeks) were purchased from Beijing Vital River Laboratory Animal Technology Co., Ltd. (Beijing, China). Mice were housed in cages, and water and food were provided *ad libitum*. All mice were allowed to acclimatize themselves for 1 week before the initiation of experiment. All protocols in this study were approved by the Research Ethics Committee of Institute of Basic Theory of Chinese Medicine, China Academy of Chinese Medical Sciences.

### 2.2. Induction of Collagen-Induced Arthritis

Bovine type II collagen (Chondrex, Redmond, WA, USA) was emulsified in an equal volume of Freund's complete adjuvant (Chondrex, Redmond, WA, USA) to a final concentration of 1 mg/mL. On day 0, mice were subcutaneously injected with 100 *μ*L of the emulsion at the base of the tail. On day 21, the animals were given booster injections intraperitoneally with 50 *μ*L of the emulsion [7].

### 2.3. Grouping and Treatment

WB was provided and identified by Liaoning China Resources Benxi Sanyao Co., Ltd. (Liaoning, China, No. 20180205). It was prepared by dissolving in double distilled water and the sample was fully blended again prior to use. Mice were randomly divided into six groups with ten mice per group after the successful induction of CIA model: control group, model group, methotrexate group (MTX, 0.3 mg/kg/d), WB low-dose group (WB-L, 0.536 g/kg/d), WB middle-dose group (WB-M, 1.073 g/kg/d, equal to that for RA patients), and WB high-dose group (WB-H, 2.146 g/kg/d). The administration of drug started from day 28 after first immunization and lasted four weeks. WB and MTX solution were orally administered in a volume of 0.1 mL/10 g. The mice in normal group and model group were administered the same volume of double distilled water.

### 2.4. Assessment of Arthritis Severity

Arthritis severity was graded using a 5-point scale (0: normal; 1: erythema and mild swelling confined to the tarsals or ankle joints; 2: erythema and mild swelling extending from the ankle to the tarsals; 3: erythema and moderate swelling extending from the ankle to the metatarsal joints; 4: erythema and severe swelling encompass the ankle, foot, and digits, or ankylosis of the limb) [8]. The total score of each mouse was calculated as the arthritic index, with a maximum possible score of 16 (4 points × 4 paws).

### 2.5. Enzyme-Linked Immunosorbent Assay

Serum was collected after eyeball blood extraction in mice. Levels of IL-1*β*, IL-6, and TNF-*α* were detected with commercially available enzyme-linked immunosorbent assay (ELISA) kits (eBioscience, San Diego, CA, USA) according to manufacturer's instructions.

### 2.6. Histological Assessment

The dissected hind paw joints of mice were fixed in 10% formalin solution and decalcified using 10% EDTA. After dehydration, specimens were then paraffin-embedded, sectioned (5 mm thickness) for hematoxylin and eosin (H&E) staining, and observed under a light microscope. Histopathological characteristics were evaluated blindly as described previoulsy [9].

### 2.7. Immunohistochemical Staining

The sections were dewaxed and hydrated using xylene and a graded series of alcohols. Activity of endogenous peroxidase was quenched with 3% H_2_O_2_. Then the tissues were incubated with anti-IL-1*β*, anti-IL-6, and anti-TNF-*α* (Abcam, Cambridge, UK) overnight at 4°C. Final color product was developed with DAB Kit (ZSGB-BIO, Beijing, China). Later, sections were counterstained with hematoxylin (Leagene, Beijing, China). Meanwhile, PBS was used for control staining instead of primary antibodies. Immunohistochemical semiquantitative analysis was performed as previously described [10].

### 2.8. TRAP Staining

The sections were stained for TRAP using a TRAP staining kit (Sigma, St. Louis, MO, USA) according to the manufacturer's protocol. Specimens were observed by computer image analysis using the Leica Qwin image analysis software (Leica Microsystem, Germany). TRAP-positive multinucleated cells that contained more than 3 nuclei were identified as osteoclasts [11].

### 2.9. Micro-CT Analysis

The fixed hind paws were placed in a centrifuge tube with physiological saline. Micro-CT analyses were performed using 1174 compact micro-CT (Skyscan, Aartselaar, Belgium). The micro-CT analysis procedures were performed according to the international guideline.

### 2.10. Western Blotting

Analysis of the proteins extracted from ankle joints by western blotting was performed using standard methods. Equivalent amounts of protein from each sample were separated in 10% SDS-polyacrylamide gel and blotted onto a PVDF membrane. The membrane was then blocked with 5% milk (BD, Sparks, MD, USA); incubated with antibodies against OPG, RANKL, p-p65, p-IKK*α*/*β*, I*κ*B*α*, and *β*-actin (Abcam, Cambridge, UK) overnight; and then hybridized with HRP-conjugated secondary antibody for 1 h. The immunoreactive bands were visualized using an ECL system (CLINX, Shanghai, China). The relative intensities of bands were quantified using Image J.

### 2.11. Statistical Analysis

All of the data were expressed as the means ± standard deviation (SD) and analyzed using GraphPad Prism 6 software. Differences in the mean values of various groups were analyzed by using ANOVA. *P* values <0.05 were considered significant.

## 3. Results

### 3.1. WB Ameliorated Arthritis Severity of CIA Mice

**Figure 1:**
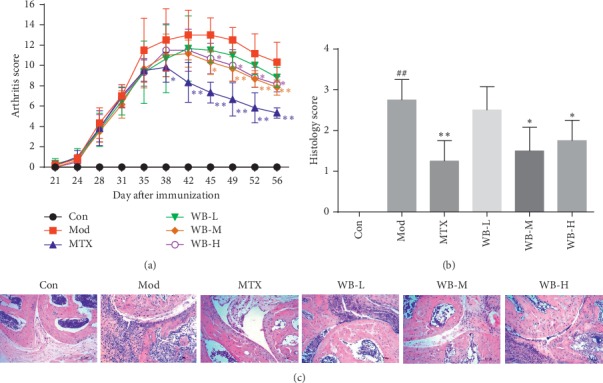
WB ameliorated arthritis severity of CIA mice. (a) Arthritis score of each group. (b) Histological score of each group. (c) Representative histological findings of ankle joint from hind paws of each group. Original magnification 200x. ^*∗*^*P* < 0.05,^*∗∗*^*P* < 0.01 vs. model group. ^##^*P* < 0.01 vs. control group.

To evaluate the therapeutic effect of WB on RA, we used CIA mice, a typical RA animal model. As shown in Figure 1(a), WB-M and WB-H treatment starting from disease onset effectively suppressed CIA progression. Significant improvement in clinical signs was observed about two weeks after WB administration, and the effect persisted until the end of the experiment. Subsequently, we analyzed the histological changes of ankle joints from the hind paws to determine the effect of WB on joint inflammation and destruction. As shown in Figures 1(b) and 1(c), the infiltration of inflammatory cells and synovial hyperplasia, as well as cartilage and bone destruction, in CIA mice were clear. WB-M and WB-H administration could alleviate those histological changes in ankle joints of CIA mice. The histological score of ankle joint was significantly lower in WB-M and WB-H treated mice than that in CIA mice.

### 3.2. WB Decreased the Levels of IL-1*β*, IL-6, and TNF-*α* in CIA Mice

**Figure 2:**
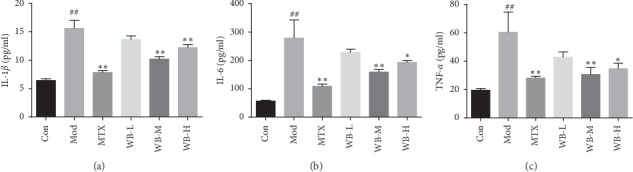
WB decreased (a) IL-1*β*, (b) IL-6, and (c) TNF-*α* levels in serum of CIA mice. Serum obtained from all group was measured by ELISA. ^*∗*^*P* < 0.05,^*∗∗*^*P* < 0.01 vs. model group. ^##^*P* < 0.01 vs. control group.

**Figure 3:**
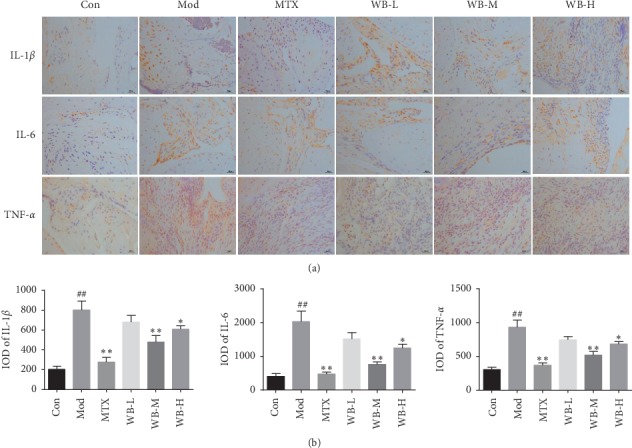
(a) Representative immunohistochemistry images of IL-1α, IL-6, and TNF-α in each group. (b) IOD means of each group. Synovium tissue sections from ankle joints in each group were stained with anti-IL-1*β*, anti-IL-6, and anti-TNF-*α*. Original magnification 400x. ^*∗*^*P* < 0.05,^*∗∗*^*P* < 0.01 vs. model group. ^##^*P* < 0.01 vs. control group.

Multiple proinflammatory cytokines, such as IL-1*β*, IL-6, and TNF-*α*, not only induce and deteriorate inflammation but also cause cartilage damage and bone destruction in RA. We therefore detected the concentrations of these proinflammatory cytokines in serum and joints of CIA mice treated with WB. As shown in Figure 2, the serum levels of IL-1*β*, IL-6, and TNF-*α* in CIA mice were obviously increased, whereas WB treatment could significantly decrease the levels of these three cytokines. Similarly, WB treatment also lowered the levels of IL-1*β*, IL-6, and TNF-*α*, which were remarkably increased in synovium tissue sections of ankle joints from CIA mice (Figure 3).

### 3.3. WB Inhibited the Bone Destruction in CIA Mice

**Figure 4:**
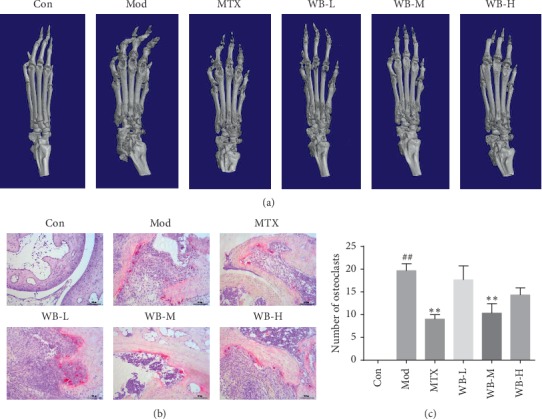
WB inhibited bone destruction in CIA mice. (a) Representative three-dimensional images of ankle joint. (b) Representative osteoclasts images of ankle joint by TRAP staining. (c) Osteoclast number of each group. ^*∗∗*^*P* < 0.01 vs. model group. ^##^*P* < 0.01 vs. control group.

Histological examination showed that WB not only alleviated the joint inflammation but also inhibited joint and bone destruction. To further investigate the effect of WB on bone destruction in CIA mice, we used micro-CT analysis and TRAP staining. As shown in Figure 4(a), the paws of the model group exhibited severe bone destruction and decreased bone density. However, paws from WB-treated groups exhibited reduced bone destruction and increased bone density, especially WB-M group. Moreover, the TRAP staining showed that the number of osteoclasts in ankle joint was remarkably higher in model group than that in control group. WB-M treatment significantly lowered the osteoclasts number in CIA mice (Figures 4(b) and 4(c)). Therefore, we used WB-M for further mechanism studies.

### 3.4. WB Regulated OPG/RANKL System

**Figure 5:**
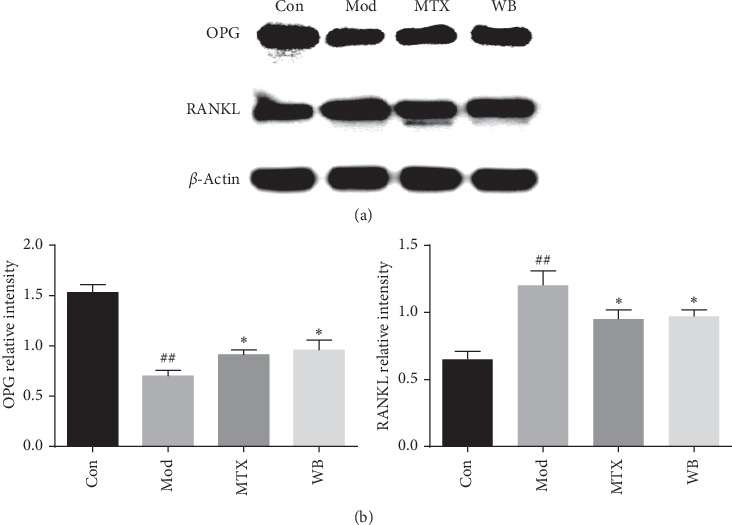
WB regulated OPG and RANKL levels in CIA mice. (a) Representative bands of western blotting in different groups. (b) Semiquantitative analysis of western blotting in different groups. ^*∗*^*P* < 0.05 vs. model group. ^##^*P* < 0.01 vs. control group.

To investigate the mechanism of WB in inhibiting bone destruction, we detected the OPG and RANKL expression in ankle joint of CIA mice by western blotting. As shown in Figure 5, The level of OPG was obviously decreased, whereas the level of RANKL was increased in model group when compared with control group. WB treatment significantly increased OPG level and, at the same time, decreased RANKL level when compared with model group.

### 3.5. WB Suppressed the Activation of NF-*κ*B Signaling Pathway

**Figure 6:**
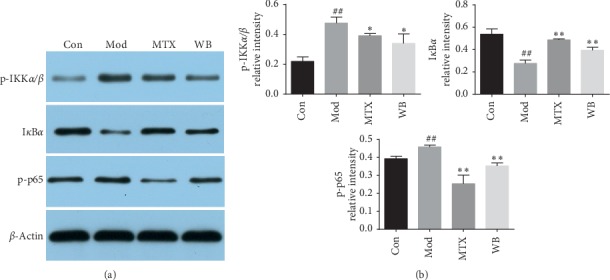
WB suppressed the activation of NF-*κ*B in CIA mice. (a) Representative bands of western blotting in different treatment groups. (b) Semiquantitative analysis of western blotting in different treatment groups. ^*∗*^*P* < 0.05,^*∗∗*^*P* < 0.01 vs. model group. ^##^*P* < 0.01 vs. control group.

The expression of p-IKK*α*/*β*, I*κ*B*α*, and p-p65 was detected by western blotting. As shown in Figure 6, WB significantly decreased the expression levels of p-IKK*α*/*β* and p-p65 in ankle joints of CIA mice. In addition, WB increased the level of I*κ*B*α* in CIA mice.

## 4. Discussion

Joint inflammation and bone destruction are the two typical pathological features of RA. In this study, we found that WB could effectively suppress the disease progression in CIA mice. Histological analysis also showed that WB treatment alleviated inflammatory cells infiltration, as well as cartilage and bone destruction in CIA mice.

Cytokines regulate a broad range of inflammatory processes in the pathogenesis of RA, especially some proinflammatory cytokines, TNF-*α*, IL-1*β*, and IL-6 [12]. TNF-*α* and IL-1*β* are central inflammatory cytokines in the pathogenesis of RA. They can exacerbate the inflammation through enhancing the release of some inflammatory mediators, including proinflammatory cytokines, chemokines, and PGE2. Simultaneously, they also participate in the bone destruction by promoting osteoclast activation [12, 13]. IL-6 was originally identified as a B cell regulatory factor, while recent findings indicate that it also acts as a regulator of CD4+ T cell proliferation, differentiation, and activation [14, 15]. Moreover, IL-6 is involved in the development of bone destruction, because it can induce osteoclast differentiation through receptor activator of NF-kappa B ligand (RANKL) expression [16]. In our study, we found that TNF-*α*, IL-1*β*, and IL-6 in serum and joint of CIA mice were significantly decreased in WB treatment group when compared with model group. These results further indicated the effectiveness of WB treatment in CIA mice.

Osteoclasts-mediated bone destruction plays key role in RA progression. Proinflammatory cytokines such as TNF-*α*, IL-1*β*, and IL-6 were reported to be osteoclastogenic; besides, interactions between receptor activator of the nuclear factor kappa B (RANK) and its ligand RANKL are essential in osteoclastogenesis. RANKL is a TNF-family cytokine required for osteoclast formation. RANK on monocyte binds to RANKL, initiating osteoclast differentiation [17, 18]. Osteoprotegerin (OPG) is another important factor participating in the regulation of osteoclast differentiation. It acts as a decoy receptor to block the effect of RANKL, thus suspending the activation of osteoclasts [19]. Regulating the OPG-RANKL system is regarded as an effective strategy to inhibit the bone destruction in RA. In our study, we found that WB treatment could not only lower the number of osteoclasts but also increase the OPG level and, at the same time, decrease the RANKL level in the joints of CIA mice, which showed its effectiveness in alleviating the bone destruction in RA.

The transcription factor NF-*κ*B is crucial in the regulation of immune responses and bone destruction in RA. It can activate I*κ*B phosphorylation by activation of I*κ*B kinase (IKK) and promote the production of p65, thereby regulating the occurrence of inflammatory responses. On the other hand, NF-*κ*B also participates in osteoclastogenesis, because it can mediate the effects of RANKL [20]. Previous studies have proved the activation of NF-*κ*B in cultured synovial fibroblasts and synovial tissue from RA patients, as well as the arthritis animal models [21]. Moreover, blocking NF-*κ*B could relieve inflammatory response and prevent bone destruction in arthritis animal models [22, 23]. Our results indicated that WB effectively inhibit the activation of NF-*κ*B, which may partly explain its effect in inhibiting the progression of joint inflammation and bone destruction.

In conclusion, this study demonstrated that WB could effectively alleviate disease progression of CIA mice by decreasing the IL-1*β*, IL-6, and TNF-*α* levels, modulating the OPG-RANKL system, and inhibiting the activation of NF-*κ*B.

## Data Availability

The data used to support the findings of this study are available from the corresponding author upon request.
